# Co-Expression of Cancer Stem Cell Markers Corresponds to a Pro-Tumorigenic Expression Profile in Pancreatic Adenocarcinoma

**DOI:** 10.1371/journal.pone.0159255

**Published:** 2016-07-14

**Authors:** Jan Skoda, Marketa Hermanova, Tomas Loja, Pavel Nemec, Jakub Neradil, Petr Karasek, Renata Veselska

**Affiliations:** 1 Laboratory of Tumor Biology, Department of Experimental Biology, Faculty of Science, Masaryk University, Brno, Czech Republic; 2 Department of Pediatric Oncology, University Hospital Brno and Faculty of Medicine, Masaryk University, Brno, Czech Republic; 3 International Clinical Research Center, St. Anne’s University Hospital and Faculty of Medicine, Masaryk University, Brno, Czech Republic; 4 1st Department of Pathological Anatomy, St. Anne’s University Hospital and Faculty of Medicine, Masaryk University, Brno, Czech Republic; 5 Department of Complex Oncology Care, Masaryk Memorial Cancer Institute, Brno, Czech Republic; Southern Illinois University School of Medicine, UNITED STATES

## Abstract

Pancreatic ductal adenocarcinoma (PDAC) remains one of the most lethal malignancies. Its dismal prognosis is often attributed to the presence of cancer stem cells (CSCs) that have been identified in PDAC using various markers. However, the co-expression of all of these markers has not yet been evaluated. Furthermore, studies that compare the expression levels of CSC markers in PDAC tumor samples and in cell lines derived directly from those tumors are lacking. Here, we analyzed the expression of putative CSC markers—CD24, CD44, epithelial cell adhesion molecule (EpCAM), CD133, and nestin—by immunofluorescence, flow cytometry and quantitative PCR in 3 PDAC-derived cell lines and by immunohistochemistry in 3 corresponding tumor samples. We showed high expression of the examined CSC markers among all of the cell lines and tumor samples, with the exception of CD24 and CD44, which were enriched under *in vitro* conditions compared with tumor tissues. The proportions of cells positive for the remaining markers were comparable to those detected in the corresponding tumors. Co-expression analysis using flow cytometry revealed that CD24^+^/CD44^+^/EpCAM^+^/CD133^+^ cells represented a significant population of the cells (range, 43 to 72%) among the cell lines. The highest proportion of CD24^+^/CD44^+^/EpCAM^+^/CD133^+^ cells was detected in the cell line derived from the tumor of a patient with the shortest survival. Using gene expression profiling, we further identified the specific pro-tumorigenic expression profile of this cell line compared with the profiles of the other two cell lines. Together, CD24^+^/CD44^+^/EpCAM^+^/CD133^+^ cells are present in PDAC cell lines derived from primary tumors, and their increased proportion corresponds with a pro-tumorigenic gene expression profile.

## Introduction

Pancreatic ductal adenocarcinoma (PDAC) is a highly lethal malignancy that represents the fourth leading cause of cancer-related deaths in Western countries [1]. PDAC has no early warning signs or symptoms; therefore, most patients present with advanced disease. The dismal prognosis of PDAC is primarily due to its late diagnosis, which is often accompanied by metastatic disease and high resistance of the primary tumor to chemotherapy and radiotherapy [2]. Despite recent advances in the diagnosis and treatment of pancreatic cancer, its incidence almost equals its mortality rate, and the 5-year survival rate does not generally reach 5% [1].

PDAC is a type of solid tumor in which transformed cells with stemness properties, termed cancer stem cells (CSCs), have been identified [3–5]. CSCs represent a subpopulation of tumor cells that can self-renew and undergo multilineage differentiation and that possess high tumorigenic potential *in vivo*. CSCs are highly resistant to conventional chemotherapy and radiotherapy and are considered a cause of tumor relapse after eradication of the tumor bulk.

The first evidence for the existence of CSCs in PDAC was reported by two groups in 2007 [3,4]. First, Li *et al*. demonstrated that the combination of cell surface markers CD44, CD24, and epithelial cell adhesion molecule (EpCAM; epithelial-specific antigen, ESA) identified a highly tumorigenic subpopulation of PDAC cells with stem cell properties [3]. Later, Hermann *et al*. reported pancreatic CSCs that were defined by the expression of prominin-1 (CD133) [4]. Since then, other putative markers of pancreatic CSCs have been found, including nestin, CXCR4, c-Met, and aldehyde dehydrogenase 1 [1, 6]. Some of these putative markers were also tested in combination with those first described. For example, c-Met^high^ cells were found to be more tumorigenic if they co-expressed CD44 [7]. CD133^+^/CXCR4^+^ cells were reported to have increased migration ability *in vitro*, and they also demonstrated metastatic potential in a mouse model [4]. However, a comprehensive study that has evaluated the co-expression of CD44, CD24, EpCAM and CD133 has not yet been conducted. Although Hermann *et al*. noted a 14% overlap among CD44^+^/CD24^+^/EpCAM^+^ and CD133^+^ cell populations in their pioneering study, this result was obtained in only one pancreatic cell line that was derived from a metastatic tumor and not from a primary tumor [4]. Similar to other combinations of CSC markers, the CD24^+^/CD44^+^/EpCAM^+^/CD133^+^ phenotype might more accurately identify true pancreatic CSCs. Thus, in the first step, the possible overlap among CD24^+^/CD44^+^/EpCAM^+^ and CD133^+^ cell populations in cell lines derived from primary PDAC should be determined. Additionally, it remains unknown to what extent the expression levels of CSC markers change under *in vitro* conditions because no study has compared the expression levels of CSC markers in PDAC tumor samples and in cell lines derived directly from those tumors.

Therefore, we performed a detailed expression analysis of the most frequently discussed putative markers of CSCs in PDAC (i.e., CD24, CD44, EpCAM, CD133, and nestin) in both human primary tumor samples and in the respective cell lines derived from those tumors. For the first time, we also examined the co-expression of CD24, CD44, EpCAM, and CD133 in cell lines derived from primary PDACs. Furthermore, these cell lines were subjected to expression profiling analysis to identify genes, the functions of which may correlate with the presence of CSC markers. We found that CD24^+^/CD44^+^/EpCAM^+^/CD133^+^ cells represented a significant subpopulation in these cell lines, and their increased proportion corresponded to a pro-tumorigenic gene expression profile.

## Materials and Methods

### Primary cell lines and tumor samples

Three PDAC primary cell lines were included in this study: P6B, P28B and P34B. These cell lines were derived from tissue samples of corresponding primary tumors. These tumor samples were obtained from patients undergoing pancreatic resection surgery as a part of standard diagnostic therapeutic procedures for PDAC, and they were de-identified to comply with the Czech legal and ethical regulations governing the use of human biological material for research purposes (Act No. 372/2011 Coll. on Health Services, paragraph 81, article 4, letter a). The patients signed a written consent containing information on this issue. Resection specimens were routinely processed at the department of pathology and during the gross inspection, the pathologist (MH) obtained the tumor tissue samples for a derivation of cell lines. For immunohistochemical (IHC) analysis, formalin-fixed, paraffin-embedded (FFPE) tumor tissue samples primarily taken for diagnostic purposes were used and selected by the pathologist (MH) who also performed the standard histopathological diagnostic procedures. A previously described protocol was used to generate the primary cultures [8]. A description of the cohort is provided in Table 1.

**Table 1:** Description of patient cohort and derived cell lines.

Tumor sample	Gender	Age	Diagnosis	Localization	Grade	Stage	OS	PFS	Cell line
P6	M	66	PDAC	Head	3	pT3N1M0	33	21	P6B
P28	M	49	PDAC	Head	3	pT3N0M0	9	9	P28B
P34	F	62	PDAC	Body	2	pT3N1M0	21	11	P34B

Gender: M, male; F, female. Age at the time of diagnosis: years. Localization: Head, head of the pancreas; Body, body of the pancreas. Grade: 2, moderately differentiated; 3, poorly differentiated. OS, overall-free survival: months. PFS, progression free survival: months.

### Cell cultures

The cell lines were cultured in DMEM supplemented with 20% fetal calf serum, 2 mM glutamine, 100 IU/ml penicillin, and 100 μg/ml streptomycin (all purchased from GE Healthcare Europe GmbH, Freiburg, Germany). The cells were maintained under standard conditions at 37°C in an atmosphere containing 5% CO_2_ and were subcultured one or two times per week.

### Immunohistochemistry

IHC detection was performed on FFPE samples of primary tumors, as mentioned above. Sections that were cut at a thickness of 4 μm were applied to positively charged slides, deparaffinized in xylene and rehydrated through a graded alcohol series. Antigen retrieval was performed in a calibrated pressure chamber Pascal (Dako, Glostrup, Denmark) for each antibody as follows: for nestin and CD133, the sections were heated in Tris/EDTA buffer (Dako) at pH 9.0 for 40 min at 97°C; for CD24, CD44 and EpCAM, the sections were heated in citrate buffer (Dako) at pH 6.1 for 4 min at 117°C. Endogenous peroxidase activity was quenched with 3% hydrogen peroxide in methanol for 20 min, followed by incubation at room temperature (RT) with the primary antibody (S1 Table). For nestin, the Vectastain Elite ABC kit using a streptavidin-biotin horseradish peroxidase (HRP) detection method was used (Vector Laboratories, Burlingame, CA, USA). For CD133 and EpCAM, the EnVision+ Dual Link system-HRP without avidin or biotin was used for detection (Dako). The expression of CD44 was visualized using an EXPOSE Rabbit-specific HRP/DAB detection kit (Abcam, Cambridge, UK), while the expression of CD24 was visualized using an ImmunoCruz ABC Staining system (Santa Cruz Biotechnology, Inc., Dallas, TX, USA). 3,3´-diaminobenzidine (DAB) (Dako) was used as the chromogen. Samples that were incubated without the primary antibodies served as negative controls. CD133- and nestin-positive endothelial cells in the tumor tissue samples served as internal positive controls, while glioblastoma multiforme tissue served as an external positive control for nestin. For EpCAM, CD44 and CD24, colon carcinoma, urinary bladder tissue and lymph node tissue, respectively, served as the positive controls. An evaluation of all IHC results was performed using an Olympus BX51 microscope and an Olympus DP72 camera with uniform settings. All immunostained slides were evaluated at 400× magnification.

### Immunofluorescence

Indirect immunofluorescence (IF) was performed as previously described [9]. The primary and secondary antibodies that were used in these experiments are listed in S1 Table; a mouse monoclonal anti-α-tubulin served as the positive control. An Olympus BX-51 microscope was used for sample evaluation; micrographs were captured using an Olympus DP72 CCD camera and were analyzed using the Cell^P imaging system (Olympus, Tokyo, Japan).

### Flow cytometry

Flow cytometry was performed on either fixed or live cells. Briefly, cells were detached from the culture flask with Accutase (Life Technologies, Carlsbad, CA, USA) and were washed in PBS. Regarding cell surface labeling, live cells were incubated in 3% BSA for 10 minutes. For both cell surface and intracellular labeling, cells were fixed in 3% paraformaldehyde (Sigma) for 30 minutes, washed twice in PBS and incubated in 3% BSA for 10 minutes. All subsequent labeling was performed at 37°C for fixed cells or at 4°C for live cells. Each sample was divided into two, and in the parallel sample, the respective isotype controls were used instead of the primary antibodies. A list of antibodies used in this study is provided in S1 Table. Briefly, the sample was washed twice with 3% BSA, incubated with the mouse monoclonal CD133 antibody for 30 minutes, and washed twice in 3% BSA. A secondary donkey anti-mouse Alexa488-conjugated antibody was applied in the same manner. After two additional washes, primary conjugated antibodies against CD24, CD44 and EpCAM were added to the sample and incubated for 30 minutes. Finally, the sample was washed four times with PBS and was subjected to analysis using FACSVerse (BD Biosciences, San Jose, CA, USA). Side scatter and forward scatter profiles were used to eliminate cell doublets. At least 10,000 events were collected per sample, and the data were analyzed using FlowJo X software (Tree Star, Inc., Ashland, OR, USA). Positive cells were evaluated relative to the respective isotype control; Boolean gating was applied to determine the cells that co-expressed the CSC markers.

### Real-Time Quantitative Reverse Transcription PCR (qRT-PCR)

Regarding qRT-PCR of PDAC cell lines, total RNA was extracted and reverse transcribed as previously described [10]. Quantitative PCR was performed in a volume of 10 μl using the KAPA SYBR^®^ FAST qPCR Kit (Kapa Biosystems, Wilmington, MA, USA) and 7500 Fast Real-Time PCR System (Applied Biosystems, Foster City, CA, USA). At least three technical replicates were analyzed for each sample. For microarray validation experiments, three biological replicates (different cell passages) of each cell line were used. The data were analyzed by 7500 Software v. 2.0.6 (Applied Biosystems) and relative quantification (RQ) of gene expression was calculated using 2^−ΔΔCT^ method [11]; heat shock protein gene (*HSP90AB1*) was used as the endogenous reference control. The primer sequences used are listed in S2 Table.

### Gene expression profiling

Total RNA was extracted using the GenElute^™^ Mammalian Total RNA Miniprep Kit (Sigma-Aldrich; St. Louis, MO, USA). Total RNA with a purity ratio of 260/280>1.7 and an integrity (RIN)>7.5 (as measured by an Agilent 2010 Bioanalyzer; Agilent Technologies, Santa Clara, CA, USA) was transcribed into cDNA (Ambion WT Expression Kit), labeled and hybridized to the Affymetrix GeneChip^®^ Human Gene ST 1.0 array and processed through a complete Affymetrix workflow (all from Affymetrix, Santa Clara, CA, USA). Raw microarray data are available in the ArrayExpress database (www.ebi.ac.uk/arrayexpress) under accession number E-MTAB-4055. Affymetrix power tools were used to normalize raw CEL files at the gene level. Robust multiarray averaging (RMA) normalization and complete annotation files were selected. Gene ontology analysis was performed using the GOTERM_BP_FAT database in the DAVID functional annotation tool [12, 13]. Cytoscape v. 3.1.1 [14] with the Reactome Functional Interaction (FI) plug-in was used for functional protein interaction network analysis. The Reactome FI plug-in gene set analysis tool was selected to include interactions from the Reactome FI network 2013 version and FI annotations.

### Statistical analysis

The qRT-PCR validation data were analyzed using one-tailed Mann-Whitney U test. *P* < 0.01 was considered statistically significant.

## Results

### CSC markers were highly expressed in PDAC-derived cell lines compared with PDAC tumor tissues

**Fig 1:**
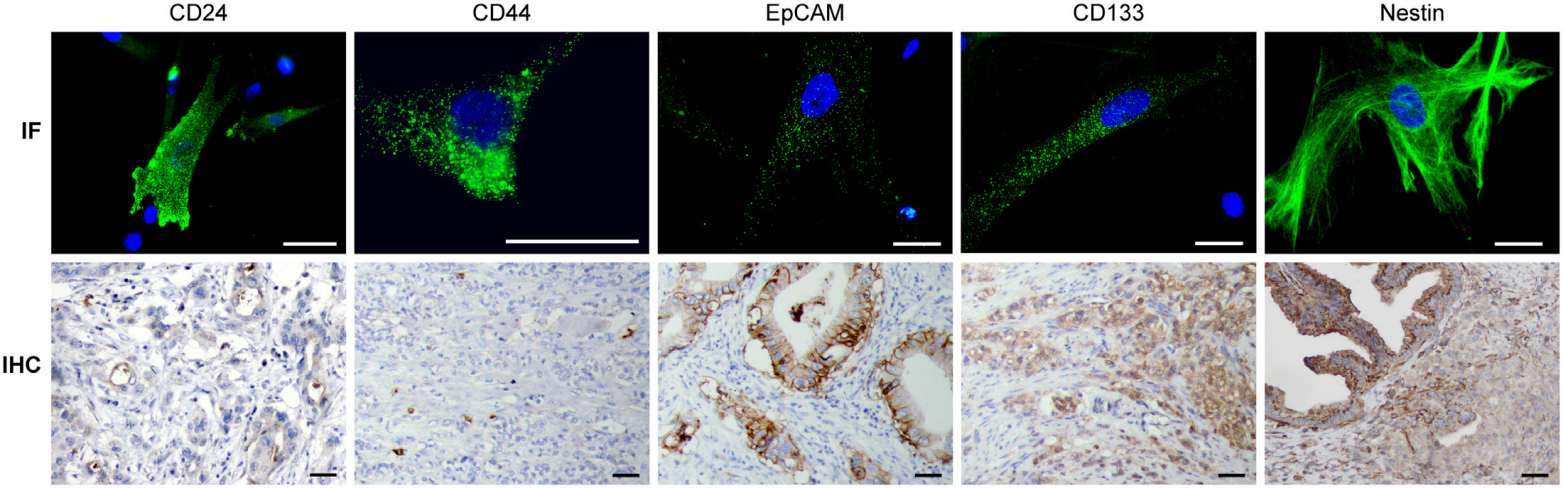
IF and IHC analysis of CSC marker expression in PDAC cell lines and corresponding tumors. Representative images of immunofluorescence (IF) and immunohistochemical (IHC) detection of CD24, CD44, EpCAM, CD133, and nestin expression are shown. For IF analysis, the cells of each PDAC cell line were stained with the appropriate antibodies against the CSC markers (green) and were counterstained with DAPI (blue) to visualize the nuclei. IHC was performed on tumor samples with antibodies that recognize specific markers; positive cells were visualized by DAB staining. Scale bars, 40 μm.

To address the expression of the putative CSC markers CD24, CD44, EpCAM, CD133, and nestin in pancreatic cancer, we used three cell lines (P6B, P28B, and P34B) derived from PDAC tumor tissues and three corresponding FFPE tumor samples (Table 1). Initially, the expression of individual CSC markers in the cell lines was assessed by IF (Fig 1). Using this method, the expression of all of the examined CSC markers was determined in all three cell lines. The observed pattern of expression of each marker was in accordance with the expected cellular localization of these molecules (Fig 1). Subsequently, the exact quantification of the proportion of cells that were positive for these markers was performed solely by flow cytometry (see below), with the exception of nestin. This was because approximately 95% of the cells in all three cell lines were nestin-positive as detected by IF; thus, nestin was omitted from the flow cytometric analysis. IHC was used to evaluate the expression levels of the CSC markers in the corresponding FFPE tumor samples (Fig 1; Table 2). IHC revealed a high percentage of tumor cells that expressed nestin and EpCAM in all of the tumor samples. In addition, CD133 was highly expressed in P6 and P28 tumors, although only a small number of positive cells was identified in P34 tumor tissue. Similarly, CD24 was expressed solely in P6 and P28 tumors. By contrast, CD44^+^ cells were identified in a poorly differentiated component of P28 tumor tissue but not in the other two tumor samples. Based on the IHC results, the P28 tumor was the only one that expressed all of the tested CSC markers.

**Table 2:** IHC analysis of CSC marker expression in PDAC tumor samples.

	Positive cells^a^			Localization of marker expression		
	P6	P28	P34	P6	P28	P34
**CD24**	++	+	-	apical cytoplasmic, luminal	apical cytoplasmic	–
**CD44**	-	+/-	-	–	poorly differentiated component	–
**EpCAM**	+++	+++	+++	membranous	membranous	membranous
**Nestin**	+++	+++	+++	cytoplasmic	cytoplasmic	cytoplasmic
**CD133**	++	+++	+	cytoplasmic	cytoplasmic, rarely membranous	cytoplasmic

^a^The percentage of positive tumor cells was categorized into five levels:—(0%), +/- (1–5%), + (6–20%), ++ (21–60%), and +++ (61–100%).

**Fig 2:**
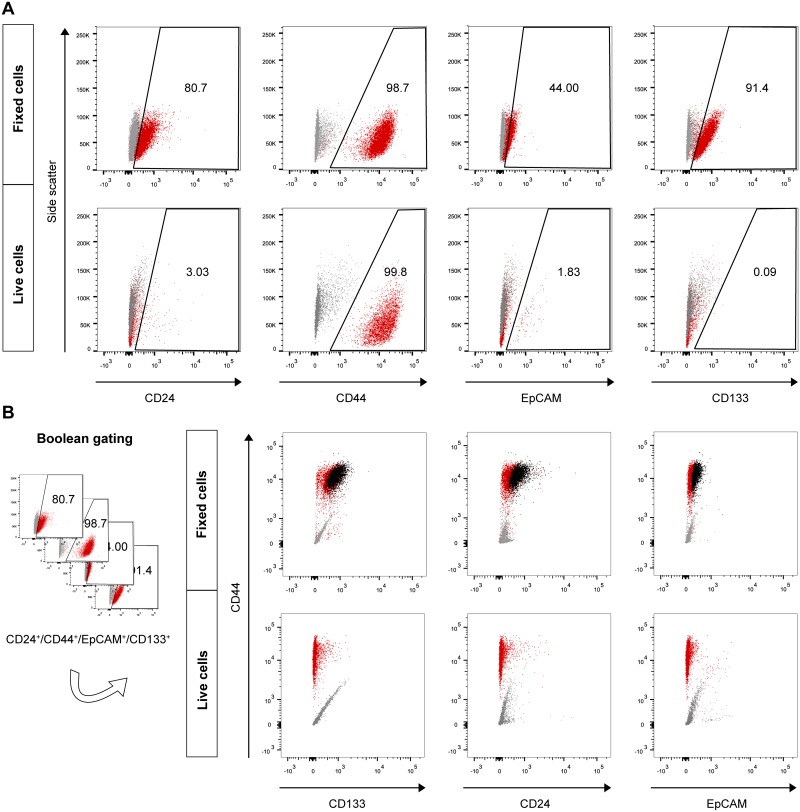
Flow cytometric analysis of the expression of CSC markers in fixed and live PDAC cells. (A) Dot plot diagrams depict the differences in CSC marker expression in PDAC cells when fixed or live cells were used in the flow cytometric analysis. The percentages of cells that were positive for specific markers are marked by numbers in the gated areas. (B) A Boolean gating approach was used to determine the proportion of cells that co-expressed CSC markers. An illustrative Boolean gate of the CD24^+^/CD44^+^/EpCAM^+^/CD133^+^ population (black) is shown in the dot plot diagrams. Cells stained with matched isotype control antibodies (gray) were used as controls for each CSC marker antibody (red) in both experimental designs (fixed cells and live cells). Representative data for the P6B cell line are shown. For detailed results of CSC marker expression, see Table 3.

Next, multicolor flow cytometry was used to evaluate the percentage of cells that were positive for CD24, CD44, EpCAM, CD133, and their combinations in three tumor-derived cell lines (Fig 2; Table 3). For multicolor flow cytometry, we used both live cells and cells fixed in paraformaldehyde. Surprisingly, using the fixed cells, we detected very high percentages of CD24^+^, CD44^+^, EpCAM^+^, and CD133^+^ cells in all of the cell lines examined (Table 3). Additionally, cells that were positive for the combinations of these markers were very common. The CD24^+^/CD44^+^/CD133^+^ phenotype was present in approximately 80% of the cells irrespective of the cell line. The percentages of CD24^+^/CD44^+^/EpCAM^+^ and CD24^+^/CD44^+^/CD133^+^/EpCAM^+^ cells varied more among the cell lines, but the percentages of each ranged from 43 to 72%. Compared with their respective tumor tissues, the cell lines were markedly enriched for CD24^+^ and CD44^+^ cells. In accordance with the IHC results, the highest frequency of the cells that expressed CD24, CD44, EpCAM, and CD133 was found in the P28B cell line.

**Table 3:** Flow cytometric analysis of CSC marker expression in PDAC cell lines.

Marker	Fixed cells^a^			Live cells^a^		
	P6B	P28B	P34B	P6B	P28B	P34B
CD24^+^	80.70	79.10	79.10	3.03	13.00	2.52
CD44^+^	98.70	96.10	96.70	99.80	98.50	98.20
EpCAM^+^	44.00	78.60	57.50	1.83	4.08	2.28
CD133^+^	91.40	94.90	89.60	0.09	0	6.70
CD24^+^/CD44^+^/EpCAM^+^	43.20	72.10	55.10	0.40	0.76	1.14
CD24^+^/CD44^+^/CD133^+^	79.70	78.10	78.00	0.06	0	1.43
CD24^+^/CD44^+^/CD133^+^/EpCAM^+^	43.20	71.90	55.00	0	0	1.14

^a^Proportions of cells positive for expression of individual CSC marker or combination of markers are indicated as percentages.

### Live cells differed greatly from fixed cells with respect to positivity for CSC markers

Due to the surprising prevalence of cells in the PDAC cell lines that were positive for CSC markers, we next used live cells for subsequent flow cytometric analyses. Because fixation itself can permeabilize the cell membranes, this approach enabled us to evaluate the expression of CSC markers only on the cell surface. Using live cells for flow cytometric analyses of the expression of CD24, CD44, EpCAM, and CD133 in the PDAC cell lines, we observed a marked decrease in positivity for these markers in compared with fixed cells (Fig 2; Table 3). In the samples of live cells, CD44 was the only marker that was detected at levels that were similar to those in fixed cell samples. However, the proportions of CD24^+^/CD44^+^/EpCAM^+^ and CD24^+^/CD44^+^/CD133^+^ cells were markedly lower and ranged from 0.4 to 1.14% and from 0 to 1.43%, respectively. CD24^+^/CD44^+^/CD133^+^/EpCAM^+^ cells were detected only in the P34B cell line.

### Gene expression profiling identified a pro-tumorigenic profile of the P28B cell line that highly co-expressed CSC markers

**Fig 3:**
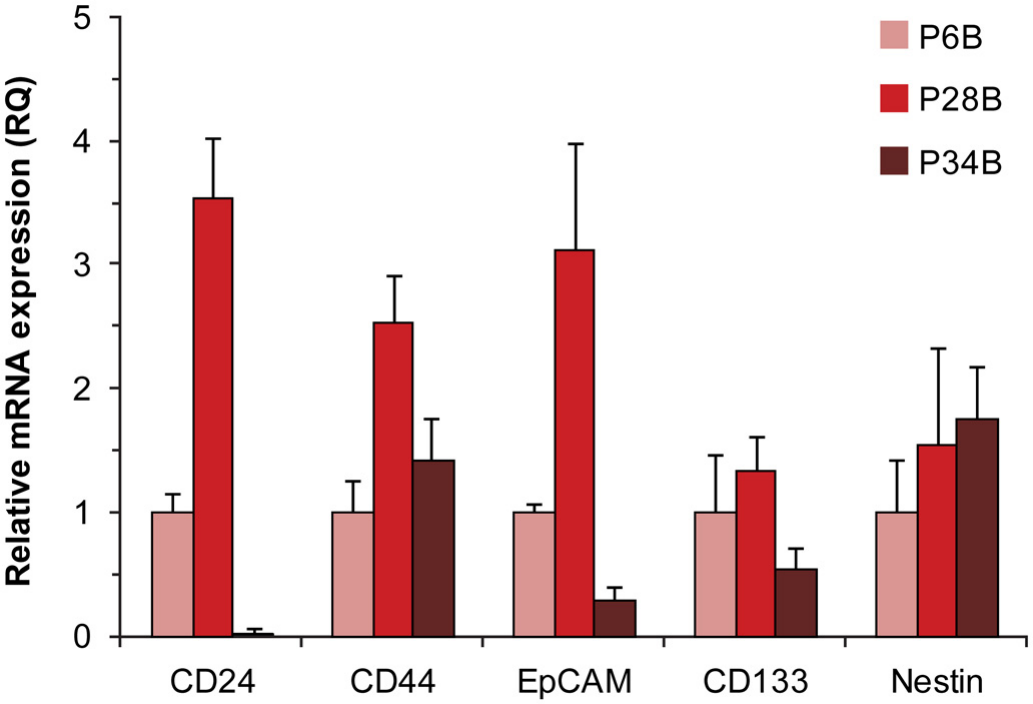
qRT-PCR analysis of CSC marker expression. P6B cell line served as the arbitrary calibrator of the gene expression. The error bars indicate the calculated maximum (RQMax) and minimum (RQMin) expression levels that represent the standard error of the mean expression level (RQ value).

To verify the expression of CSC markers observed at the protein level and investigate possible differences among the PDAC cell lines, we next evaluated gene expression at the mRNA level. In the first step, we performed qRT-PCR for the genes that encode the CSC markers (Fig 3). qRT-PCR revealed upregulated mRNA expression of the proteins CD24, CD44, and EpCAM in P28B cells. As clinical data show, the P28B cell line was derived from the tumor of the patient with the shortest overall survival (P28, Table 1). IHC, flow cytometry and qRT-PCR results all revealed that P28B cells also expressed the highest levels of CSC markers among the tested cell lines. To investigate this phenomenon more thoroughly, we employed gene expression profile analysis. Using this method, we detected 344 genes that were upregulated (fold-change ≥ 2), and 258 genes that were downregulated (fold-change ≤ 0.5) in the P28B cells compared with the expression profiles of P6B and P34B cell lines.

**Fig 4:**
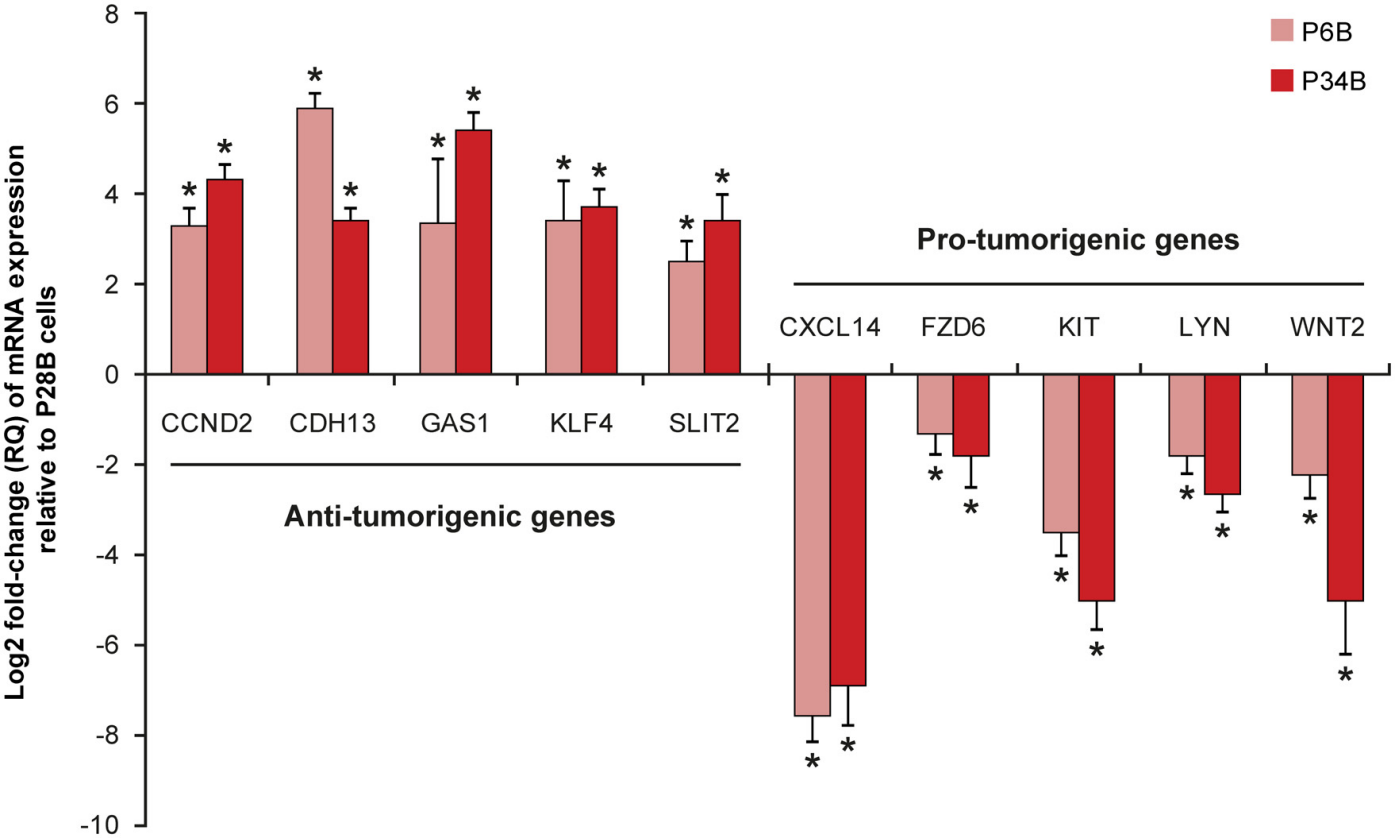
Validation of pro-tumorigenic expression profile of P28B cells by qRT-PCR. Five anti-tumorigenic and five pro-tumorigenic genes were selected based on the microarray data and their expression was validated by qRT-PCR. The graph shows the expression levels of the respective genes in P6B and P34B cells relative to that in P28B cell line, which served as the arbitrary calibrator. The bars represent the mean expression level (RQ value) of three biological replicates; the data are presented in log2 scale. The calculated maximum (RQMax) and minimum (RQMin) expression levels are indicated by error bars. *P < 0.001, indicates significant differences from P28B cell line.

To analyze the biological functions of the differentially expressed genes in P28B cells, we performed gene ontology analysis (Table 4; S3 Table). Most of the upregulated genes were found to be associated with cell surface receptor signaling (18.8% of the upregulated genes) or cell adhesion (9.3%). Downregulated genes were linked to the regulation of cell proliferation (11.2% of the downregulated genes), cell motility (7.6%) or regulation of apoptosis (7%). Furthermore, a review of the literature revealed that the vast majority of the upregulated genes have been reported to have pro-tumorigenic potential, whereas about one-third of the downregulated genes were found to suppress tumorigenesis (Table 5; S4 Table). To validate the results obtained by expression profiling, we performed qRT-PCR analysis of five pro-tumorigenic and five anti-tumorigenic genes in samples from three different passages of each cell line (Fig 4). In agreement with the microarray data, all selected anti-tumorigenic genes were significantly (*P* < 0.001) downregulated in P28B cells, whereas the expression of pro-tumorigenic genes was significantly (*P* < 0.001) upregulated compared with that of P6B and P34B cells. Taken together, these analyses revealed a specific pro-tumorigenic profile of the P28B cell line that was highly enriched in cells that co-expressed CSC markers. By contrast, the lower expression of CSC markers in the P6B and P34B cell lines reflected differences in the expression profiles of these cell lines compared with the profile of P28B cells.

**Table 4:** Gene ontology analysis of genes differentially expressed in P28B cells.

Biological process	Number of genes	P value
*Upregulated genes (fold-change ≥ 2)*		
Cell surface receptor linked signal transduction	65	< 0.001
Cell adhesion	32	< 0.001
G-protein coupled receptor protein signaling pathway	30	0.049
Ion transport	29	< 0.001
Cell-cell signaling	28	< 0.001
Regulation of cell proliferation	26	0.007
Response to wounding	22	0.001
Immune response	20	0.061
Cell motion	16	0.034
*Downregulated genes (fold-change ≤ 0*.*5)*		
Regulation of cell proliferation	29	< 0.001
Cell motion	20	< 0.001
Regulation of apoptosis	18	0.039
Immune response	17	0.022
Cell adhesion	17	0.024
Mitotic cell cycle	11	0.026
Vasculature development	10	0.006

Upregulated (fold-change ≥ 2) and downregulated (fold-change ≤ 0.5) genes in P28B cells compared with P6B and P34B cells were analyzed for gene ontology. Gene ontology analysis was performed using GOTERM_BP_FAT database in DAVID functional annotation tool.

**Table 5:** Differentially expressed genes in P28B cells grouped by their role in tumorigenesis.

Role in cancer	Number of genes	Genes
*Upregulated genes (fold-change ≥ 2)*		
Pro-tumorigenic	62	ABCC4, ADAMTS7, ADM, ANO1, BAMBI, CD24, CP, CSF1, CXCL14, CXCR7, CYP1A1, EDN1, ELMO1, ENTPD1, EPHA6, F3, FGFR4, FZD6, FZD7, GFRA1, GPR183, GPR56, GPR65, GRIA4, CHRM3, IL6R, ITGB3, JAM2, KIT, LAMA3, LPAR3, LYN, MCAM, MITF, NCAM2, NLK, NOG, NOX4, P2RY1, PMP22, PREX2, PTGER4, PTHLH, RPS6KA5, SCN5A, SEMA4D, SEMA6A, SHC3, SLC4A4, SMAD9, SORT1, TEK, TFAP2C, TRPA1, TRPC3, TRPC6, TRPV2, UCP2, VTN, WFDC1, WNT2, WNT2B
Anti-tumorigenic	10	DSC2, DSC3, FOXF1, GBP2, PENK, PPAP2A, RELN, RGS6, TNFSF10, TXNIP
Mixed	9	ADAMTS8, CD9, DSG2, F11R, CHL1, ITGA8, NPY, SMURF2, UNC5C
*Downregulated genes (fold-change ≤ 0*.*5)*		
Pro-tumorigenic	28	ADRA2A, ARHGEF2, BGN, CENPF, CTSS, DLGAP5, ENPP2, GLI3, HORMAD1, CHST11, IL1A, IL1B, IL6, JAG1, KCNMA1, MET, MSX2, NFIB, NRP1, PLAUR, PLK1, PTX3, SEMA3C, SERPINE1, SPOCK1, TPBG, VASH2, VCAN
Anti-tumorigenic	18	CCND2, CDH13, CLDN11, EMILIN2, EPHB2, GAS1, CHST11, KLF4, KYNU, NEFL, PCDH10, PLA2G4A, RARB, SERPINB2, SLIT2, SRPX, TGFBR3, UNC5B
Mixed	16	ASPM, BUB1B, CD74, CDC6, CDKN3, CLU, CTH, ENPEP, FYN, ITGA2, ITGA3, POSTN, PRRX1, RAC2, TOP2A, UACA

The role of individual genes in tumorigenesis was determined based on the literature review (S4 Table).

**Fig 5:**
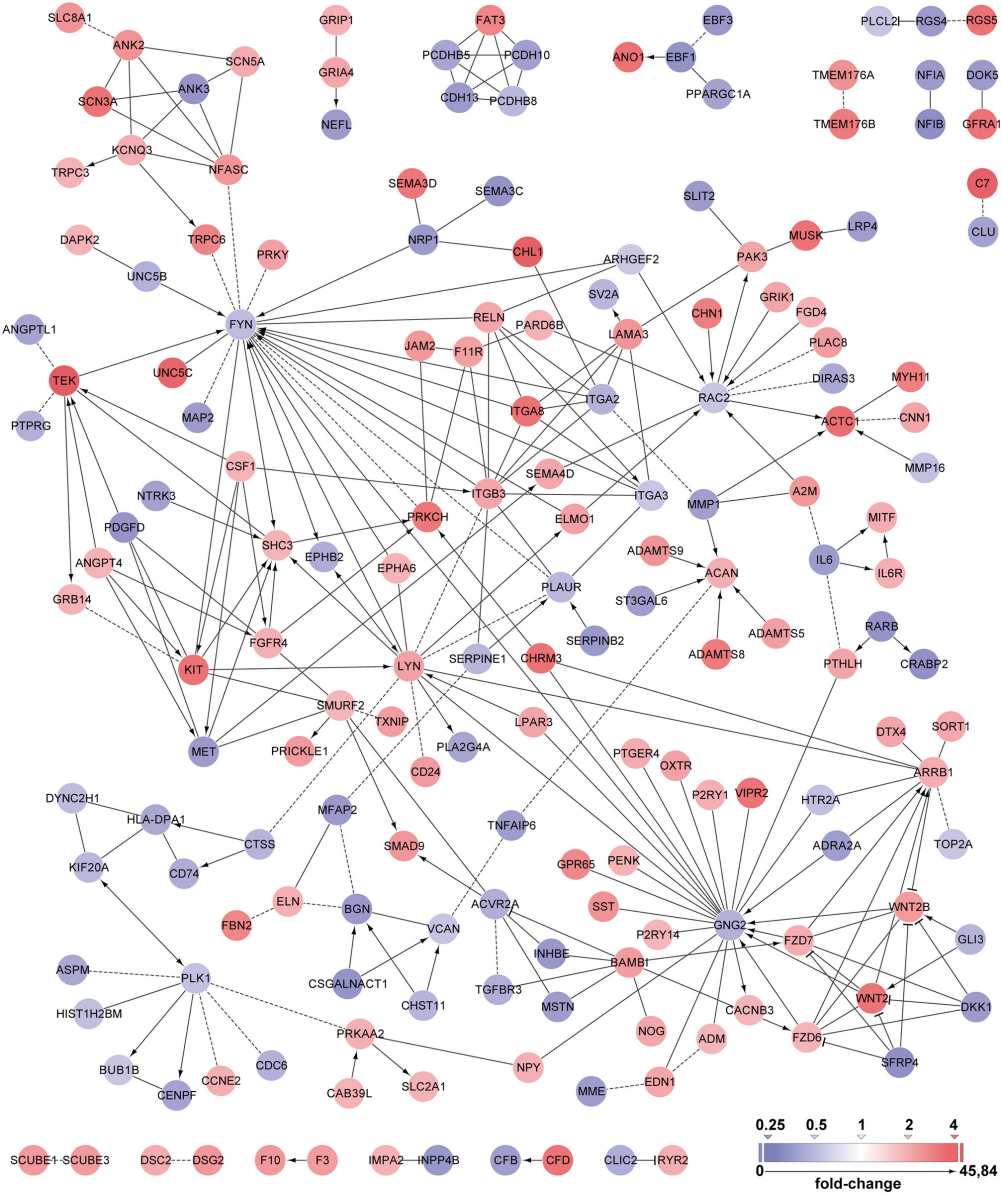
Functional protein network analysis based on a set of differentially expressed genes in P28B cells. A set of upregulated (fold-change ≥ 2) and downregulated (fold-change ≤ 0.5) genes in P28B cells compared with P6B and P34B cells was visualized with Cytoscape. A Reactome FI plug-in was used to analyze the functional network of proteins that are encoded by the respective genes. The fold-change values of gene expression are depicted as tints of blue (downregulated genes) or red (upregulated genes) color.

To further analyze the expression profile of P28B cells compared with that of P6B and P34B cells, we performed a functional protein interaction network analysis of the differentially expressed genes. Using Cytoscape software with the Reactome FI plug-in, we created an interaction network that enabled us to visualize expression profiling data combined with information on the interactions of the proteins encoded by the respective genes (Fig 5). This approach clearly showed the most prominent genes whose expression was upregulated or downregulated in P28B cells compared with the other two cell lines. Downregulated genes included *FYN*, *RAC2*, *GNG2*, *PLK1* and *MET*. Of the upregulated genes, *LYN*, *WNT2*, *KIT*, *TEK* (*TIE2*) and *ARRB1* were identified.

Together, our results indicated that a high proportion of cells that expressed CSC markers corresponded with a pro-tumorigenic expression profile. In addition, the highest expression of CSC markers was found in the tumor sample taken from the patient with the shortest survival and also in the P28B cell line that was derived from this tumor.

## Discussion

Although pancreatic CSCs were described nearly ten years ago as CD44^+^/ CD24^+^/EpCAM^+^ cells [3] or CD133^+^ cells [4], no study has determined the co-expression of all of these markers in PDAC either directly in the tumor samples or in the human PDAC cell lines derived from primary tumors. Therefore, the present study was focused on a detailed analysis of the expression of putative CSC markers (CD24, CD44, EpCAM, CD133 and nestin) in 3 pairs of matched primary PDAC tissue samples and derived cell lines.

We detected the expression of all of the examined markers in each tumor cell line. Markedly high levels of nestin were detected in all cell lines and corresponding tumors. Because nestin was expressed in most of the cells, these results suggest that nestin is not suitable as a CSC marker in PDAC, a finding that is in accordance with the results of our previous study [15]. Therefore, we omitted nestin from further flow cytometric co-expression analyses. In the cell lines, flow cytometric analysis of fixed cells revealed a high proportion of cells that expressed CSC markers. IHC confirmed that the expression patterns of the CSC markers were similar in the corresponding tumor tissues, although CD24 and CD44 expression levels were considerably lower. An increased proportion of CD24^+^ and CD44^+^ cells in PDAC cell lines compared with the original tumor tissues might indicate that these cells had a selective advantage in cell culture. This finding is in agreement with other studies that reported high percentages of CD44^+^ cells in pancreatic cell lines compared with PDAC tumor tissues [16, 17]. However, in these studies, the cell lines that were used were not derived from the examined tumors; therefore, it is difficult to determine the baseline expression levels of these markers in the original tumor tissues for comparison. Our study is the first to show that the proportion of CD44^+^ and/or CD24^+^ cells increases in PDAC-derived cell lines compared with the corresponding tumor tissues. This phenomenon should be considered when performing *in vitro* studies of CSCs in PDAC.

Surprisingly, flow cytometric analysis of fixed cells showed that CD24, CD44, CD133, or EpCAM, as evaluated separately, are expressed in more than 80% of cells irrespective of the cell line and marker (except EpCAM in P6B and P34B cells). These values are much higher than those that have been previously reported [16, 17]. However, when live cells were used in the flow cytometry experiments, we detected a significant decrease in the proportion of CD24^+^, CD133^+^ and EpCAM^+^ cells (Table 3). Using this approach, the levels of positivity for each of these markers were comparable to those in the aforementioned studies [16, 17]. Only CD44 expression in live cells was detected at the same level as in fixed cells (Table 3). Nevertheless, the reason for this discrepancy is obvious. It is widely known that fixation with paraformaldehyde permeabilizes the cell membranes and therefore enables the antibody to bind to the proteins that are localized within the cell. By contrast, live cells have intact membranes, and antibodies can bind only to extracellular epitopes of the proteins. This means that when fixed cells were used for flow cytometry, we could also detect cells that expressed the CSC markers within the cell.

It was previously thought that most CSC marker proteins performed their functions at the cell surface. However, growing evidence has indicated that the subcellular localization of CSC markers can vary greatly, possibly leading to completely different effects of these proteins on cell signaling, proliferation, invasiveness, and metastatic potential. Therefore, the distinct subcellular localization of CSC markers may result in different patient outcomes. For example, several studies have reported cytoplasmic localization of CD24 in PDAC [18, 19] as well as in other tumor types [20–27]. Cytoplasmic CD24 expression has been identified as a marker of poor prognosis in gastric cancer [25], colorectal cancer [22, 23], ovarian cancer [20, 21] and malignant neoplasms of the salivary glands [26]. However, little is known concerning the functional role of CD24 within the cell. It has been reported that intracellular CD24 may inhibit the invasiveness of PDAC cells [19]. Nevertheless, a recently published study showed that intracellular CD24 promotes the growth of prostate cancer cells through the inhibition of p14ARF, resulting in decreased levels of p53 and p21 [27]. In that study, the authors also reported that CD24 positivity increased substantially when detection was performed with fixed cells. Recently, very similar findings have also been shown in breast cancer [28]. These observations are in agreement with our results and indicate that a significant amount of CD24 protein may be located in the cytoplasm of PDAC cells.

CD133 is another marker that we examined, and the cell surface immunoreactivity of this protein was significantly lower than the intracellular immunoreactivity. Originally, CD133 was introduced as a marker of pancreatic CSCs that is expressed in approximately 2% of PDAC cells [4]. Several flow cytometric studies then reported a similar low proportion of CD133^+^ cells (0–28%) in PDAC [16, 17]. However, these results are in contrast with the high (up to 100%) CD133 positivity of cells detected by IHC even in the aforementioned studies [16, 17, 29]. IHC analysis also demonstrated that a significant amount of CD133 was localized within the cytoplasm of PDAC cells [29], a finding that is in agreement with our results (Tables 2 and 3). We and other groups have recently shown that membranous localization of CD133 may be altered in tumor cells and that intracellular CD133 may be involved in cell signaling pathways [9, 30–33]. Furthermore, the correlation of high intracellular CD133 expression with poor prognosis has been found in different types of tumors [33–36]. The results of the present study note the need for better understanding of the role of the intracellular expression of CSC markers in PDAC. Considering that flow cytometric analyses in previously published studies were typically performed to detect cell surface expression in live cells, these studies might have significantly underestimated the expression levels of CD24 (a maximum of 30% CD24^+^ cells were reported [3, 16, 17]) and CD133 in PDAC cells, which may lead to misinterpretation of the results as discussed by other authors [37].

In the present study, we showed for the first time that cells co-expressing CD24, CD44, EpCAM, and CD133 are present in human PDAC cell lines derived from primary tumors. Moreover, CD24^+^/CD44^+^/EpCAM^+^/CD133^+^ cells represented a significant population of cells (range, 43.2 to 71.9%) among the cell lines. By contrast, the proportion of cells that co-expressed these markers at the cell surface was very limited (range, 0 to 1.43%) as indicated by flow cytometry with live cells (Table 3). These differences in subcellular localization represent a practical restriction in the isolation of CD24/CD44/EpCAM/CD133-positive and -negative cell populations. Sorting the cells based on cell surface labeling alone could be problematic because a large proportion of cells that express CSC markers within the cell would be sorted into negative fractions, likely compromising the results of further experiments. In a recent comprehensive study, Huang *et al*. reported that both CSC marker-positive (CSC^+^) and -negative (CSC^−^) populations of cells could initiate tumors in immunodeficient mice [38]. For various tumor types, they showed that not only were CSC^+^ cells able to produce CSC^−^cells but CSC^−^cells could produce CSC^+^ cells over long-term period in culture. These results suggested that tumorigenic cells might not be able to be distinguished by common CSC markers due to the phenotypic plasticity of tumor cells. However, the expression of CSC markers was evaluated only by flow cytometry followed by cell sorting. Because the authors used only live (non-permeabilized) cells in their experiments, they might have overlooked the cells that expressed CSC markers localized in the cytoplasm or cell nucleus. This might also explain why the expression of CSC markers was detected in CSC^−^cells by PCR. We speculate that the shift of CSC marker proteins from the cytoplasm to the plasma membrane and vice versa could, to a certain extent, explain the phenotypic plasticity of the FACS-sorted cells observed by Huang *et al*. and other groups [38–40]. Nevertheless, we suggest that the detection of CSC markers located within the cell should be included in future studies to validate and extend the data that are based solely on cell surface expression.

Our results revealed that the proportion of CD24^+^/CD44^+^/EpCAM^+^/CD133^+^ cells differed among the cell lines and that the highest number of cells that co-expressed all of these markers was detected in the P28B cell line, which was derived from the tumor of the patient with the shortest overall survival. Therefore, we decided to further analyze the differences among the cell lines using gene expression profiling to identify genes that may be associated with high expression levels of CSC markers. For this reason, the expression profile of P28B cells was compared with the profiles of P6B and P34B cells. Gene ontology analysis and a review of the literature revealed a specific pro-tumorigenic expression profile of P28B cells (Table 5; S4 Table). As high tumorigenic potential is a widely accepted hallmark of CSCs, this result clearly corresponds to the increased proportion of cells that co-express CSC markers in the P28B cell line. However, it should be noted that the pro-tumorigenic expression profile of P28B cells does not imply stemness of CD24^+^/CD44^+^/EpCAM^+^/CD133^+^ cells and subsequent functional *in vivo* assays are needed to determine whether CD24^+^/CD44^+^/EpCAM^+^/CD133^+^ phenotype specifically identifies PDAC cells which fulfill all the criteria defining CSCs. Of the 602 differentially expressed genes in P28B cells, the 10 most prominent genes were identified using functional protein network analysis. These genes could represent potential targets in PDAC because their expression was associated with the co-expression of CSC markers.

Fyn and Lyn are non-receptor tyrosine kinases that belong to the Src family. It has been reported that *LYN* expression is downregulated during embryonic stem cell differentiation, whereas *FYN* expression remains constant [41]. Lyn facilitates glioblastoma cell survival [42], and *LYN* expression is associated with migration and invasion in breast cancer [43]. In a study on pancreatic cancer, the downregulation of Lyn kinase activity reduced invasiveness and migration of the cells [44]. In the present study, we found that *LYN* expression was notably upregulated in P28B cells. In a colorectal cancer study, Su *et al*. reported that the overexpression of CD24 promoted cancer cell invasion through the activation of Lyn and its interaction with Erk1/2 [45]. Patients whose tumors had a lower expression of CD24 or Lyn had a higher survival rate. In accordance with these results, we showed the upregulation of *CD24* and *LYN* in P28B cells, which were derived from the tumors of patient with the shortest overall survival. This indicates that the overexpression of the CD24/Lyn axis might also play a role in PDAC. By contrast, the expression of Fyn kinase was downregulated in P28B cells. The overexpression of Fyn has been detected in various cancers, but its role in cancer is controversial [46–48]. Fyn has been reported to correlate with the metastasis of PDAC, while the inhibition of Fyn decreased liver metastasis in nude mice [47]. By contrast, the expression of Fyn kinase induces the differentiation and growth arrest of neuroblastoma cells [46]. Moreover, Fyn is downregulated in advanced tumor stages, and its downregulation predicts the short-term survival of patients with neuroblastoma. This is in agreement with our results where the downregulation of Fyn was observed in the P28B cell line. However, the exact role of Fyn kinase in PDAC has yet to be determined.

Of the other downregulated genes in P28B cells, *GNG2* was the most prominent. This gene encodes the Gγ2 subunit that forms Gβγ dimers of heterotrimeric G proteins [49]. Although it was reported that the overexpression of GNG2 inhibits the migration and invasiveness of melanoma cells [50], little is known about the function of *GNG2* in PDAC or in other tumor types. Our study presents the first evidence that the downregulation of *GNG2* is associated with CD24^+^/CD44^+^/EpCAM^+^/CD133^+^ cells and might indicate a poor prognosis in patients with PDAC.

Recently, Yu *et al*. published a study that analyzed the expression profiles of circulating pancreatic tumor cells [51]. They determined that the expression of *WNT2* was upregulated in these cells. Their additional functional experiments showed that Wnt2 promotes anchorage-independent cell survival and the metastatic potential of pancreatic cancer cells. These results are in accordance with our findings as follows: *WNT2* was overexpressed in the P28B cell line, which contains the highest proportion of cells that express CSC markers and is derived from the tumor of the patient with the shortest overall survival. Moreover, expression profiling revealed that inhibitors of Wnt (i.e., DKK1 [52] and SFRP4 [53]) were downregulated in P28B cells compared with the other two cell lines. We also showed the upregulation of WNT2B, FZD7 and FZD6, which are other components of the Wnt signaling pathway. Recently, WNT2B was found to correlate with poor prognosis in PDAC [54], FZD6 expression was reported to be a marker of tumorigenic stem-like cells [55], and FZD7 was required for the maintenance of an undifferentiated phenotype of embryonic stem cells [56]. The upregulation of these genes in P28B cells indicates that the Wnt pathway was activated in cells that were highly positive for CSC markers. These results support the hypothesis that Wnt pathway signaling is of high importance in PDAC tumorigenesis [57].

## Conclusions

Our study showed that putative CSC markers (i.e., CD24, CD44, EpCAM, CD133, and nestin) are highly expressed in PDAC. Although the expression of these markers was enhanced in PDAC-derived cell lines, the expression pattern of each individual cell line corresponded to that of the original corresponding tumor specimen. We demonstrated that a large proportion of cells expressed some typically membranous CSC markers (i.e., CD24, EpCAM and CD133) solely within the cell. Thus, these proteins may also play other currently unknown roles in the cytoplasm of PDAC cells, and further research is necessary to determine the biological significance of this finding. Most importantly, our study is the first to show that CD24^+^/CD44^+^/EpCAM^+^/CD133^+^ cells are present in human PDAC cell lines derived from primary tumors. Although CD24^+^/CD44^+^/EpCAM^+^/CD133^+^ cells were common under *in vitro* conditions, we showed that a higher proportion of these cells in the PDAC cell line corresponded with a pro-tumorigenic gene expression profile. Upregulated Wnt signaling, upregulated expression of *LYN*, and downregulation of *FYN* expression were primarily associated with the proportion of cells that co-expressed CSC markers. In summary, these results suggest that CD24^+^/CD44^+^/EpCAM^+^/CD133^+^ cells may be of further interest in the research of PDAC and emphasize the need for further studies that would investigate whether CD24^+^/CD44^+^/EpCAM^+^/CD133^+^ phenotype specifically identifies pancreatic CSCs.

## Supporting Information

S1 Table Primary, conjugated primary and secondary antibodies used in this study. (PDF)

S2 Table Primer sequences used for qRT-PCR. (PDF)

S3 Table List of upregulated and downregulated genes in P28B cells according to the gene ontology analysis. (PDF)

S4 Table The role of differentially expressed genes in tumorigenesis—review of literature. (PDF)
