# Supplementary material for: Co-Expression of Cancer Stem Cell Markers Corresponds to a Pro-Tumorigenic Expression Profile in Pancreatic Adenocarcinoma
Source: PLoS One. 2016 Jul 14;11(7):e0159255. doi: 10.1371/journal.pone.0159255 (PMC4945008; doi:10.1371/journal.pone.0159255)
Supplement: S1 Table — (PDF) [file pone.0159255.s001.pdf]

S1 Table. Primary, conjugated primary and secondary antibodies used in this study.

**Primary antibodies**						
Antigen	Host / Type	Clone	Manufacturer	Dilution		
				IHC	IF	FC
CD24	Goat / Poly		Santa Cruz Biotechnology	1 : 50	1 : 50	-
CD44	Rabbit / Mono	EPR1013Y	Abcam	1 : 50	1 : 100	-
EpCAM	Mouse / Mono	B302 (323-A3)	Abcam	1 : 50	1 : 50	-
CD133	Mouse / Mono	17A6.1	Millipore	1 : 100	1 : 100	1 : 100
Nestin	Mouse / Mono	10C2	Millipore	1 : 200	1 : 200	-
α-tubulin	Mouse / Mono	TU-01	Exbio	-	1 : 100	-
**Conjugated primary antibodies**						
Antigen	Host	Conjugate	Manufacturer	Dilution		
				IHC	IF	FC
CD24	Mouse	PE/Cy7	Biolegend	-	-	1 : 20
CD44	Mouse	Brilliant Violet 421	Biolegend	-	-	1 : 20
EpCAM	Mouse	APC	Biolegend	-	-	1 : 20
**Secondary antibodies**						
Host	Specifity	Conjugate	Manufacturer	Dilution		
				IHC	IF	FC
Donkey	anti-Rabbit IgG	Alexa Fluor 488	Invitrogen	-	1 : 200	-
Donkey	anti-Mouse IgG	Alexa Fluor 488	Invitrogen	-	1 : 200	-
Donkey	anti-Goat IgG	Alexa Fluor 488	Invitrogen	-	1 : 200	-

Mono, monoclonal; Poly, polyclonal.
