# Supplementary material for: Co-Expression of Cancer Stem Cell Markers Corresponds to a Pro-Tumorigenic Expression Profile in Pancreatic Adenocarcinoma
Source: PLoS One. 2016 Jul 14;11(7):e0159255. doi: 10.1371/journal.pone.0159255 (PMC4945008; doi:10.1371/journal.pone.0159255)
Supplement: S2 Table — (PDF) [file pone.0159255.s002.pdf]

S2 Table. Primer sequences used for qRT-PCR.

Gene symbol	Gene full name	Forward primer (5'→3')	Reverse primer (5'→3')
CCND2	Cyclin D2	GACTGAGCTGCTGGCTAAGAT	CTTGGATCCGTCACGTTGGT
CD24	CD24 molecule	GCTCCTACCCACGCAGATTTA	GACCACGAAGAGACTGGCTG
CD44	CD44 molecule (Indian blood group)	CCTGGCAGCCCCGATTATTT	AAGGACACACCCAAGCAAGG
CDH13	Cadherin 13	GCAGGCAATTTCACCACCAA	AAATGCAGGTTGGTGCCCAT
CXCL14	C-X-C motif chemokine ligand 14	AGCACTTTTTACCAACGGTCAG	TGGAGCACAAGAGAGATGGG
EPCAM	Epithelial cell adhesion molecule	TGCTGGAATTGTTGTGCTGG	AAGATGTCTTCGTCCCACGC
FZD6	Frizzled class receptor 6	GTATGAAAATGGCCTACAACATGAC	TCCAGATTTGCGAGAGGAAGAA
GAS1	Growth arrest specific 1	CTCGTCTGCCGTCCAGAAAG	GCACCTTCCCTTTCGAGTCC
KIT	KIT proto-oncogene receptor tyrosine kinase	ACAATGGCACGGTTGAATGT	CAGGGTGTGGGGATGGATTT
KLF4	Kruppel like factor 4	ATCTTTCTCCACGTTCGCGTCTG	AAGCACTGGGGGAAGTCGCTTC
LYN	LYN proto-oncogene, Src family tyrosine kinase	CCAGGGAGGAGCCCATTTAC	AAGCAGCACTTTGCCACCTT
PROM1	Prominin 1 (CD133)	CCATTGACTTCTTGGTGCTGT	TGGAGTTACGCAGGTTTCTCT
NES	Nestin	AGTGATGCCCCTTCACCTTG	GCTCGCTCTCTACTTTCCCC
SLIT2	Slit guidance ligand 2	TGACCAACGGACCAATGACC	CATGGCCCTCCAAGCACTTA
WNT2	Wnt family member 2	TGGCTCCCTCTGCTCTTGAC	CTGGCACATTATCGCACATCAC
HSP90AB1	Heat shock protein 90kDa alpha (cytosolic), class B member 1	CGCATGAAGGAGACACAGAA	TCCCATCAAATTCCTTGAGC
