# Supplementary material for: Co-Expression of Cancer Stem Cell Markers Corresponds to a Pro-Tumorigenic Expression Profile in Pancreatic Adenocarcinoma
Source: PLoS One. 2016 Jul 14;11(7):e0159255. doi: 10.1371/journal.pone.0159255 (PMC4945008; doi:10.1371/journal.pone.0159255)
Supplement: S3 Table — (PDF) [file pone.0159255.s003.pdf]

S3 Table. List of upregulated and downregulated genes in P28B cells according to gene ontology analysis

Gene ontology	Set of genes
Upregulated genes (fold-change ≥ 2)	
GO:0007166~cell surface receptor linked signal transduction	**NOG**, GRIK1, **PREX2**, **EDN1**, **MITF**, **LPAR3**, VIPR2, **WNT2**, FOXF1, **CXCR7**, LBP, **SHC3**, CFD, CIB2, **LYN**, **PTGER4**, **PTHLH**, **ADAMTS7**, **CHRM3**, **ADM**, **GPR56**, **SORT1**, OR10K2, **GPR183**, TWSG1, **FGFR4**, USP9Y, GPR63, **GPR65**, OXTR, CACNB3, **ITGB3**, **KIT**, GPRC5B, ADAP1, MUSK, **TEK**, **P2RY1**, **CD24**, D4S234E, PPAP2A, **ENTPD1**, DTX4, TXNIP, GABRA2, **SMAD9**, **NLK**, **GRIA4**, **FZD7**, **FZD6**, **WNT2B**, **RPS6KA5**, **SEMA6A**, **LAMA3**, **EPHA6**, PENK, NPY*, ITGA8*, P2RY14, RGS6, **GFRA1**, SMURF2*, RGS9, **BAMBI**, SST
GO:0007155~cell adhesion	PPFIA2, **VTN**, **ITGB3**, **CDH6**, **CD9**, FAT3, FAT4, **TEK**, ACAN, **SSX2IP**, **CD24**, **ENTPD1**, APBA1, F11R*, SVEP1, **EGFL6**, SDK1, NFASC, CLDN22, **MCAM**, **NCAM2**, **LAMA3**, NLGN4Y, DSG2*, ITGA8*, **GPR56**, DSC3, DSC2, RELN, **SEMA4D**, **JAM2**, CHL1*
GO:0007186~G-protein coupled receptor protein signaling pathway	**GPR183**, GPR63, **EDN1**, **PREX2**, **GPR65**, **LPAR3**, OXTR, GPRC5B, VIPR2, **CXCR7**, **P2RY1**, PPAP2A, **ENTPD1**, D4S234E, GABRA2, **PTGER4**, **FZD7**, **FZD6**, **PTHLH**, NPY*, PENK, **ADM**, **CHRM3**, P2RY14, **GPR56**, RGS6, **SORT1**, RGS9, SST, OR10K2
GO:0006811~ion transport	SLC38A4, SCN3A, GRIK1, **TRPV2**, **ANO1**, CCL8, SLC38A11, CACNB3, KCNK12, **WNT2**, KCNQ3, **SLC4A4**, **SCN5A**, CNNM2, GABRA2, KCND3, SLC8A1, **TRPC3**, **TRPC6**, **TRPA1**, **GRIA4**, CNNM1, P2RX5, NPY*, **UCP2**, **ABCC4**, RYR2, KCNH7, **CP**
GO:0007267~cell-cell signaling	**FGFR4**, GRIK1, **EDN1**, **LPAR3**, CCL8, OXTR, VIPR2, **WNT2**, NPTX1, MUSK, KCNQ3, **TEK**, **CD24**, **SHC3**, APBA1, GABRA2, **LYN**, **GRIA4**, **PTHLH**, CCL13, TNFSF10, **CXCL14**, NPY*, **ADM**, **GPR56**, **TFAP2C**, **PMP22**, SST
GO:0042127~regulation of cell proliferation	**FGFR4**, **NOG**, **CSF1**, **EDN1**, **MITF**, **KIT**, FLT3LG, **WNT2**, CD9*, **TEK**, **CD24**, PPAP2A, NKX2-3, TXNIP, **NOX4**, **LYN**, **IL6R**, **PTHLH**, ADAMTS8*, **ADM**, **F3**, **WFDC1**, **PMP22**, SST, MAB21L2, ICOSLG
GO:0009611~response to wounding	**NOX4**, F11R*, C7, A2M, **NOG**, F10, **CYP1A1**, **LYN**, SCUBE1, CCL8, **IL6R**, **ITGB3**, IGSF10, CD9*, CCL13, **ADM**, **F3**, **P2RY1**, **CD24**, LBP, CFD, **ENTPD1**
GO:0006955~immune response	**GPR183**, C7, **PTGER4**, **LYN**, **GPR65**, CCL8, **VTN**, **IL6R**, CCL13, TNFSF10, SARM1, **CXCL14**, P2RY14, **SEMA4D**, LBP, **CD24**, CFD, ICOSLG, GBP2, NKX2-3
GO:0006928~cell motion	PALM, MDGA1, NFASC, **IL6R**, **KIT**, DNAH5, **ELMO1**, **WNT2**, CD9*, **SEMA6A**, NPY*, RELN, **CD24**, UNC5C*, PPAP2A, NKX2-3
Downregulated genes (fold-change ≤ 0.5)	
GO:0042127~regulation of cell proliferation	**NRP1**, CLU*, PRRX1*, **JAG1**, PNP, **GLI3**, **MSX2**, RAC2*, **SERPINE1**, **CHST11**, **ADRA2A**, **IL1B**, RARB, **IL1A**, CDC6*, **ARHGEF2**, **IL6**, ITGA2*, GAS1, CDKN3*, **VASH2**, CDH13, PLA2G4A, CTH*, CCND2, TGFBR3, KLF4, **NFIB**, HTR2A
GO:0006928~cell motion	**IL6**, **NRP1**, **ENPP2**, **MET**, ITGA2*, **SPOCK1**, GAS1, ENPEP*, SLIT2, **PLAUR**, EPHB2, CDH13, UNC5B, ANK3, FYN*, **ADRA2A**, **IL1B**, TGFBR3, **SEMA3C**, **VCAN**
GO:0042981~regulation of apoptosis	**KCNMA1**, **ARHGEF2**, **IL6**, CLU*, GAS1, **GLI3**, CD74*, **MSX2**, CDH13, PLA2G4A, UACA*, **CHST11**, SERPINB2, **IL1B**, RARB, TOP2A*, NEFL, **IL1A**
GO:0006955~immune response	IL18R1, KYNU, **IL6**, **ENPP2**, PRG4, CFB, CLU*, IFI44L, **CTSS**, PNP, CD74*, **IL1B**, TGFBR3, **SEMA3C**, HLA-DPA1, **PTX3**, **IL1A**
GO:0007155~cell adhesion	**NRP1**, PCDHB8, PCDHB5, PCDH10, ITGA2*, **SPOCK1**, ITGA3*, POSTN*, CLDN11, ECM2, EMILIN2, **TPBG**, CDH13, TNFAIP6, PGM5, SRPX, **VCAN**
GO:0000278~mitotic cell cycle	CDC6*, **ARHGEF2**, CCND2, **PLK1**, NCAPG, **DLGAP5**, BUB1B*, **CENPF**, **HORMAD1**, CDKN3*, ASPM*
GO:0001944~vasculature development	CDH13, **BGN**, **NRP1**, PRRX1*, TGFBR3, **IL1B**, **SEMA3C**, ENPEP*, **JAG1**, SLIT2

Pro-tumorigenic genes (bold), anti-tumorigenic genes (underlined) and genes with both effects in tumorigenesis (asterisk, *) are indicated based on literature review (see S4 Table).
