# Supplementary material for: Co-Expression of Cancer Stem Cell Markers Corresponds to a Pro-Tumorigenic Expression Profile in Pancreatic Adenocarcinoma
Source: PLoS One. 2016 Jul 14;11(7):e0159255. doi: 10.1371/journal.pone.0159255 (PMC4945008; doi:10.1371/journal.pone.0159255)
Supplement: S4 Table — (PDF) [file pone.0159255.s004.pdf]

S4 Table. The role of differentially expressed genes in tumorigenesis – review of literature.

Gene	Role in cancer	References (PMID)
ABCC4	pro-tumorigenic	21989485
ADAMTS7	pro-tumorigenic	18927302
ADAMTS8	mixed	16152618; 25636539
ADM	pro-tumorigenic	25475159; 12606950
ADRA2A	pro-tumorigenic	18416462; 18604234; 24559182
ANO1	pro-tumorigenic	24493744; 24639373
ARHGEF2	pro-tumorigenic	24525234; 22847784
ASPM	mixed	24830737; 23896173; 21923303
BAMBI	pro-tumorigenic	19328798; 20189233; 24912656; 23807684
BGN	pro-tumorigenic	23007878; 21879307; 24228112
BUB1B	mixed	23242215; 23154965
CCND2	anti-tumorigenic	12684418; 22303414; 17270028
CD24	pro-tumorigenic	17283135; 22335271
CD74	mixed	24594996; 24463084; 19499276; 22611320
CD9	mixed	22095071; 25955689; 15047125; 20940407
CDC6	mixed	18048387; 12006585; 16572177; 22201124
CDH13	anti-tumorigenic	20607704; 15245595
CDKN3	mixed	23775190; 25735390; 24573179; 22390936
CENPF	pro-tumorigenic	17205517; 23791740; 23163484; 20828406
CLDN11	anti-tumorigenic	21468549; 19956721; 24999589
CLU	mixed	12370533; 22967941; 21609464
CP	pro-tumorigenic	25595436
CSF1	pro-tumorigenic	24498570; 11257139
CTH	mixed	15347670; 25205294; 22360859; 25193114
CTSS	pro-tumorigenic	24875536; 23629809; 25086747
CXCL14	pro-tumorigenic	18054154; 23294544
CXCR7	pro-tumorigenic	25407240; 25341042
CYP1A1	pro-tumorigenic	23576571; 19531241
DLGAP5	pro-tumorigenic	24324629; 22022601; 24349376
DSC2	anti-tumorigenic	23836524; 25119898
DSC3	anti-tumorigenic	22941060; 24664224; 21364582
DSG2	mixed	24166502; 4896103; 19091121
EDN1	pro-tumorigenic	18758494
ELMO1	pro-tumorigenic	25360637; 23591873; 24819662; 22415709
EMILIN2	anti-tumorigenic	24374807; 20360940
ENPEP	mixed	24885240; 14998491
ENPP2	pro-tumorigenic	19855166; 12119361
ENTPD1	pro-tumorigenic	22751118; 21390184
EPHA6	pro-tumorigenic	26041887
EPHB2	anti-tumorigenic	20339854; 24096486; 24959213
F11R	mixed	25416560; 25916097; 24265754; 25033702
F3	pro-tumorigenic	18373885
FGFR4	pro-tumorigenic	12447688; 25031272; 23344261; 26045670
FOXF1	anti-tumorigenic	24186199
FYN	mixed	20151426; 19968749; 12450793
FZD6	pro-tumorigenic	22249030
FZD7	pro-tumorigenic	24474766
GAS1	anti-tumorigenic	22846196; 22311470; 21111449

Gene	Role in cancer	References (PMID)
GBP2	anti-tumorigenic	23001506; 19003964
GFRA1	pro-tumorigenic	23351331; 25009298
GLI3	pro-tumorigenic	23176625; 20814245
GPR183	pro-tumorigenic	25852561
GPR56	pro-tumorigenic	17575113
GPR65	pro-tumorigenic	23707809
GRIA4	pro-tumorigenic	21207374
HORMAD1	pro-tumorigenic	18089785; 22776561
CHL1	mixed	21408220
CHRM3	pro-tumorigenic	22222710
CHST11	pro-tumorigenic	26084610; 21658254
CHST11	anti-tumorigenic	25586191; 21658254
IL1A	pro-tumorigenic	17096856
IL1B	pro-tumorigenic	17096856
IL6	pro-tumorigenic	24097820
IL6R	pro-tumorigenic	26130650; 22552503
ITGA2	mixed	24500968; 26258411; 25662931
ITGA3	mixed	23652300; 9508191; 23786209
ITGA8	mixed	15592496; 20678483,
ITGB3	pro-tumorigenic	21622897; 21996748
JAG1	pro-tumorigenic	25309874
JAM2	pro-tumorigenic	23277282
KCNMA1	pro-tumorigenic	22899999; 17146446
KIT	pro-tumorigenic	21320746
KLF4	anti-tumorigenic	25060774; 25137052; 24060862
KYNU	anti-tumorigenic	24064976
LAMA3	pro-tumorigenic	23907728; 19945619
LPAR3	pro-tumorigenic	20331961; 22876164
LYN	pro-tumorigenic	17088984; 22805580
MCAM	pro-tumorigenic	21286374; 25685061; 22754372
MET	pro-tumorigenic	21864475
MITF	pro-tumorigenic	26168401; 25605940
MSX2	pro-tumorigenic	23162473
NCAM2	pro-tumorigenic	21214674
NEFL	anti-tumorigenic	25312269; 22319610; 2399247
NFIB	pro-tumorigenic	21764851; 23161775
NLK	pro-tumorigenic	26269673; 25371216; 24789020
NOG	pro-tumorigenic	22547073; 21249149
NOX4	pro-tumorigenic	21118808; 24946933
NPY	mixed	21439311
NRP1	pro-tumorigenic	22025255
P2RY1	pro-tumorigenic	23090120
PCDH10	anti-tumorigenic	25590240; 24740680; 25086586; 20353276
PENK	anti-tumorigenic	24289328
PLA2G4A	anti-tumorigenic	23307260
PLAUR	pro-tumorigenic	25310970; 23843896
PLK1	pro-tumorigenic	22892842; 15141022
PMP22	pro-tumorigenic	21159173; 15995147; 20847343
POSTN	mixed	25840689
PPAP2A	anti-tumorigenic	25210149

Gene	Role in cancer	References (PMID)
PREX2	pro-tumorigenic	23753921; 25151370; 24375644
PRRX1	mixed	23201163; 23807160; 25404478; 25428393
PTGER4	pro-tumorigenic	23090667
PTHLH	pro-tumorigenic	11893937
PTX3	pro-tumorigenic	25964543; 23828517; 24457902
RAC2	mixed	20822528; 24770346; 21893191
RARB	anti-tumorigenic	25232244; 24720764
RELN	anti-tumorigenic	16472607; 20847288
RGS6	anti-tumorigenic	25120791; 23598467
RPS6KA5	pro-tumorigenic	23604116; 21106525
SCN5A	pro-tumorigenic	20651255; 22678159
SEMA3C	pro-tumorigenic	22924992; 25464848
SEMA4D	pro-tumorigenic	21812859; 20858260
SEMA6A	pro-tumorigenic	25082288; 23007403; 25576923
SERPINB2	anti-tumorigenic	18548086
SERPINE1	pro-tumorigenic	26180080
SHC3	pro-tumorigenic	22311806; 15870690
SLC4A4	pro-tumorigenic	25612232
SLIT2	anti-tumorigenic	20068157; 23314850
SMAD9	pro-tumorigenic	22452883
SMURF2	mixed	25191523; 22231558
SORT1	pro-tumorigenic	25871389; 25037567
SPOCK1	pro-tumorigenic	23022495; 24134845
SRPX	anti-tumorigenic	15021917; 19424611; 12874760; 12716466
TEK	pro-tumorigenic	18366015
TFAP2C	pro-tumorigenic	24469049
TGFBR3	anti-tumorigenic	17999987; 17192875; 23387308
TNFSF10	anti-tumorigenic	24948009
TOP2A	mixed	25237769; 25304659; 11943709
TPBG	pro-tumorigenic	25066861
TRPA1	pro-tumorigenic	24037916; 23219522
TRPC3	pro-tumorigenic	23580589
TRPC6	pro-tumorigenic	23497198; 24418082; 23700295; 18452628
TRPV2	pro-tumorigenic	25001513
TXNIP	anti-tumorigenic	22750447
UACA	mixed	14961764; 23204231
UCP2	pro-tumorigenic	25805929; 24523901; 22705884
UNC5B	anti-tumorigenic	21922135; 24528886
UNC5C	mixed	20032384; 23178624; 19331160
VASH2	pro-tumorigenic	25916042
VCAN	pro-tumorigenic	19160015; 23449934; 24927163
VTN	pro-tumorigenic	19998373; 23327926; 23462327
WFDC1	pro-tumorigenic	18842679; 15305342
WNT2	pro-tumorigenic	25232495; 25146168; 22763454
WNT2B	pro-tumorigenic	25120849

PMID – reference number for PubMed citations.
